# Efficacy and safety of decitabine combined with HAAG (homoharringtonine, aclarubicin, low-dose cytarabine and G-CSF) for newly diagnosed acute myeloid leukemia

**DOI:** 10.3389/fonc.2022.998884

**Published:** 2022-10-12

**Authors:** Jun-Feng Zhu, Hai-Ping Dai, Qian-Qian Zhang, Jia Yin, Zheng Li, Qin-Ya Cui, Xiao-Peng Tian, Si-Ning Liu, Zheng-Ming Jin, Xia-Ming Zhu, De-Pei Wu, Xiao-Wen Tang

**Affiliations:** ^1^ National Clinical Research Center for Hematologic Diseases, Jiangsu Institute of Hematology, The First Affiliated Hospital of Soochow University, Suzhou, China; ^2^ Institute of Blood and Marrow Transplantation, Collaborative Innovation Center of Hematology, Soochow University, Suzhou, China; ^3^ Department of Hematology, The First Affiliated Hospital of Bengbu Medical College, Bengbu, China

**Keywords:** induction chemotherapy, decitabine, HAAG, acute myeloid leukemia, efficacy and safety

## Abstract

The 7 + 3 regimen is the front-line induction chemotherapy in patients with newly diagnosed acute myeloid leukemia, with a response rate of 60-80%. But it’s not suitable for all patients especially old/unfit patients because of a higher treatment related toxicity. Therefore, safer and more effective induction therapies are required. In this retrospective study, 50 patients with newly diagnosed acute myeloid leukemia received decitabine combined with HAAG (homoharringtonine, aclarubicin, low-dose cytarabine and G-CSF) as induction chemotherapy. Complete remission (CR) rate was 96% (48/50) and overall response rate was 100%. Of note, All 7 patients harboring *FLT3-ITD* mutation achieved CR. The median overall survival (OS) was 40.0 months (range 2.0, 58.0). The OS at 1, 3, and 5 years were 75.3%, 54.2%, and 49.3%. The median relapse free survival (RFS) was 38.0 months (range 2.0, 58.0). The RFS at 1, 3, and 5 years were 67.3%, 48.9%, and 45.1%. The OS and RFS of patients who received hematopoietic stem cell transplantation (HSCT) were significantly higher than those who did not undergo HSCT (*p*=0.017; 0.016). The incidence of grade 3-4 neutropenia and thrombocytopenia was 84% and 88%. Meanwhile, the incidence of grade 3-4 infection and bleeding was only 16% and 6%. There was no early death. In conclusion, DAC+HAAG regimen is effective and well-tolerated as induction therapy in patients with newly diagnosed AML.

## Introduction

Standard therapy for acute myeloid leukemia (AML) consists of cytarabine combined with idarubicin or daunorubicin (1). This so-called “7+3” regimen results in complete remission (CR) in 60-80% of younger patients (<60 years) and 40-60% of older patients (≥ 60 years) (2). In addition, among patients with AML who received the 7 + 3 regimen, those with poor performance status or high-risk cytogenetics had a worse prognosis than those without these features (3). Therefore, less intensive but also effective regimens are needed to be explored.

Yamada et al. reported for the first time a CAG regimen containing a low-dose cytarabine, doxorubicin and G-CSF for induction therapy in AML (4). This regimen has the advantage of enhanced cytotoxicity to S-phase cells, low intensity and prolonged duration of activities, and has therefore been widely used in China and Japan to treat elderly AML patients or AML patients with myelodysplastic features (5). However, this regimen is not suitable for patients with high white blood cell counts and unfavorable cytogenetic risks.

In acute myeloid leukemia, DNA hypermethylation of gene promoters is frequently observed and is often associated with a differentiation arrest. Global hypomethylation of genes in AML patients following treatment with a DNA methyltransferase inhibitor, leading to reactivation of differentiation capacity (6). Hypomethylating agents such as decitabine exert antileukemic effects due to its immunomodulatory properties. Hypomethylated drugs such as decitabine exert anti-leukemia effects due to their immunomodulatory properties. A study shows that decitabine upregulates tumor-associated antigen expression, promotes the induction of specific T cell responses, and makes AML cells more susceptible to NK cell-mediated killing (7). Decitabine has been used successfully in AML (8), but the CR rate of decitabine monotherapy is only 27% (9). Therefore, in recent years, decitabine combined with CAG regimen has been tried in elderly acute myeloid leukemia (10). Homoharringtonine is a plant alkaloid that was first isolated from Cephalotaxus in China. The anti-leukemic effects of homoharringtonine are primarily based on the inhibition of protein synthesis, thereby inducing leukemia cell differentiation, inhibiting proliferation, and promoting apoptosis (11, 12). Homoharringtonine also synergized with cytarabine and aclarubicin (13).

In a previous study, we incorporated decitabine into the HAAG priming regimen (homoharringtonine, aclarubicin, low-dose cytarabine, and G-CSF) in patients with refractory/relapsed AML, and achieved an overall response rate (ORR) of 83% and complete remission (CR) rate of 58% (14). It is reasonable to speculate that the DAC+HAAG regimen is also suitable for newly diagnosed AML patients. Here, we retrospective analyzed the efficacy, safety and tolerability of DAC+HAAG as induction therapy in 50 adult patients with newly diagnosed AML.

## Patients and methods

This study included newly diagnosed AML patients who received the DAC+HAAG regimen between September 1, 2014 and April 30, 2019 in the Department of Hematology, the First Affiliated Hospital of Soochow University, Suzhou, China. AML was diagnosed according to the 2016 WHO classification (15). Patients with acute promyelocytic leukemia and those had previously received combined chemotherapy and/or targeted therapy for AML were excluded. The study protocol was approved by the Ethics Committee of the First Affiliated Hospital of Soochow University. All patients provided informed consent for treatment in accordance with the Declaration of Helsinki. Patients with the following characteristics are preferentially included in the DAC+HAAG regimen: 1. ≥60 years old; 2. Low proliferative AML or AML with myelodysplasia-related changes. 3. Cardiac insufficiency, ≥ grade 2. 2. Systemic infection, ≥ grade 2. R-banding was administered for karyotypic analysis (16). Mutations were detected by sanger sequencing (5 gene panels for 2/50 patients), next generation sequencing (40 gene panels for 48/50 patients) (17). Risk stratification was evaluated according to ELN 2017 (2). Multiparamemter flow cytometry based on “different-from-normal” principle was used for MRD detection. Antigens used in detection of MRD included CD7, CD10, CD19, CD33, CD34, CD38, CD45, CD56, CD117 and HLA-DR.

### Treatments

Fifty patients were treated with DAC+HAAG regimen, which consists of decitabine 20 mg/m^2^/d intravenously on days 1-5, homoharringtonine 1 mg/d intravenously on days 3-16, aclarubicin 10 mg/d intravenously on days 3-10, cytarabine (Ara-C) 10 mg/m^2^ q12h subcutaneously on days 3-16, and granulocyte colony-stimulating factor (G-CSF) 50-300 µg/d subcutaneously on days 2-16. Consolidation chemotherapy consists of one cycle of the DAC+HAAG regimen (for those achieved CR), followed by high-dose Ara-C (3g/m^2^, over 3 hours, every 12 hours on days 1-3, IV) or the FA regimen (fludarabine, 30 mg/m^2^/d on days 1-5, IV and Ara-C, 2g/m^2^/d on days 1-5, IV) for 1-2 cycles before HSCT or 3-4 cycles for those didn’t undergo HSCT. Patients with intermediate or unfavorable risk leukemia can receive autologous or allogenic hematopoietic stem cell transplantation (HSCT) according to from American Society for Blood and Marrow Transplantation guidelines (18).

### Assessments

Treatment response was assessed according to the International Working Group criteria for AML (19). Early mortality was defined as death within 4 weeks of starting chemotherapy. OS was measured from the date of diagnosis to the date of death or last follow-up. PFS was defined as the duration from CR to relapse or death of any cause. The time to neutrophil recovery was from the end of chemotherapy to the first day when neutrophil count recovered to ≥ 0.5×10^9^/L measured on two consecutive days. The time to platelet recovery was measured from the end of chemotherapy to the first day that platelet count recovered to ≥ 20×10^9^/L (for at least 7 consecutive days). Toxicities during induction chemotherapy were graded according to the Common Terminology Criteria for Adverse Events version 5.0. All follow-up visits were conducted by review of medical records, outpatient reviews and telephone calls. Follow-up was up May 30, 2019. And continuous CR, relapse, and death were recorded at the last follow-up.

### Statistical analysis

Cox proportional hazards models were used to estimate hazard ratios (HRs) and 95% confidence intervals for the associations between prognostic factors and OS. All variables with *P* < 0.05 in univariable analysis were kept in multivariable analysis to obtain the best of predictors. Statistical analyses was performed by SPSS statistics for Windows, Version 19.0 (Armonk, NY: IBM Corp.) and R software (version 4.0.3; http://www.Rproject.org).

## Results

### Patient demographic and baseline characteristics

**Figure 1:**
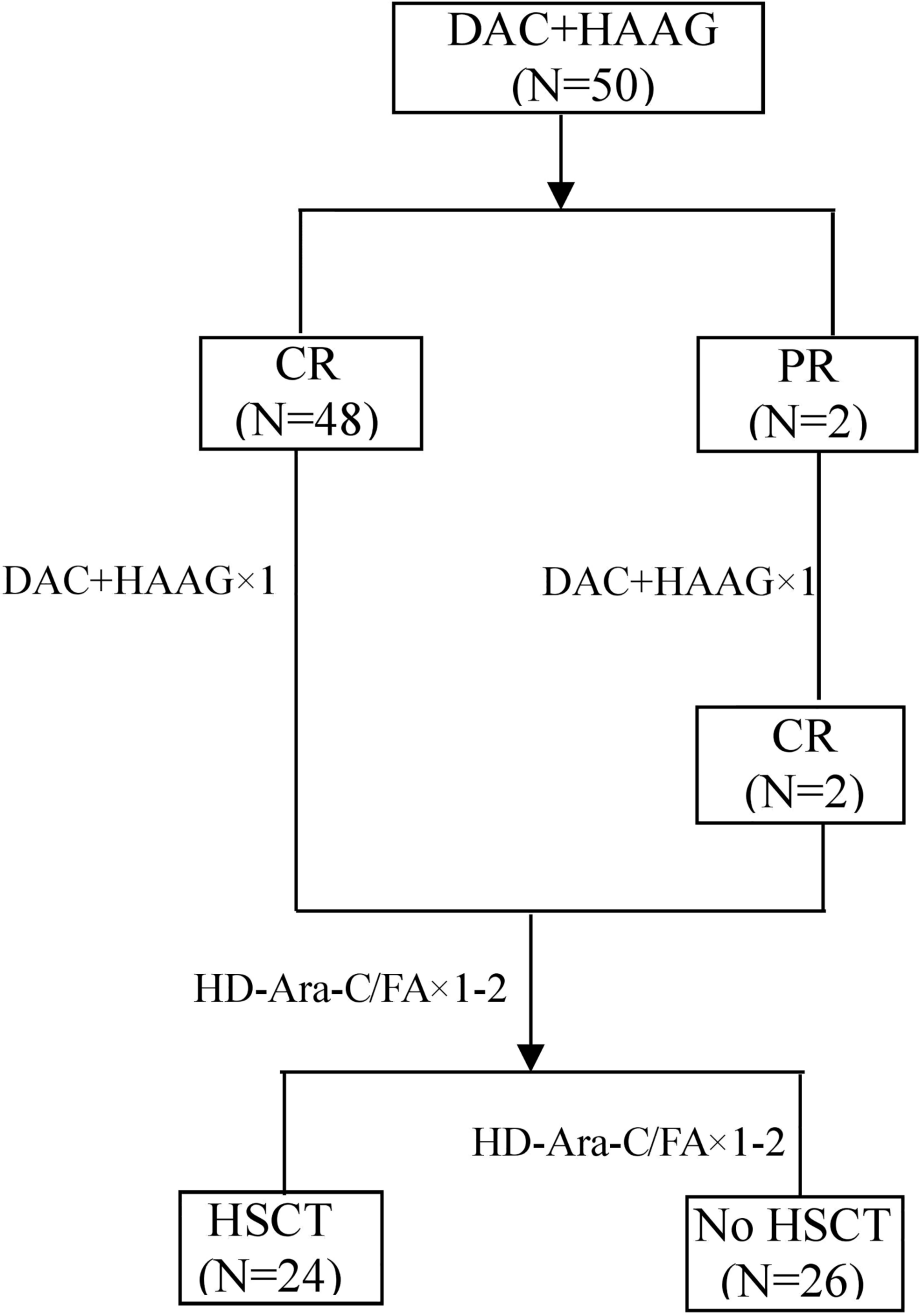
The study flow chart. CR, complete remission; PR, partial remission; HD-Ara-c, high-dose cytarabine; FA, fludarabine combined with cytarabine; HSCT, hematopoietic stem cell transplantation.

**Figure 2:**
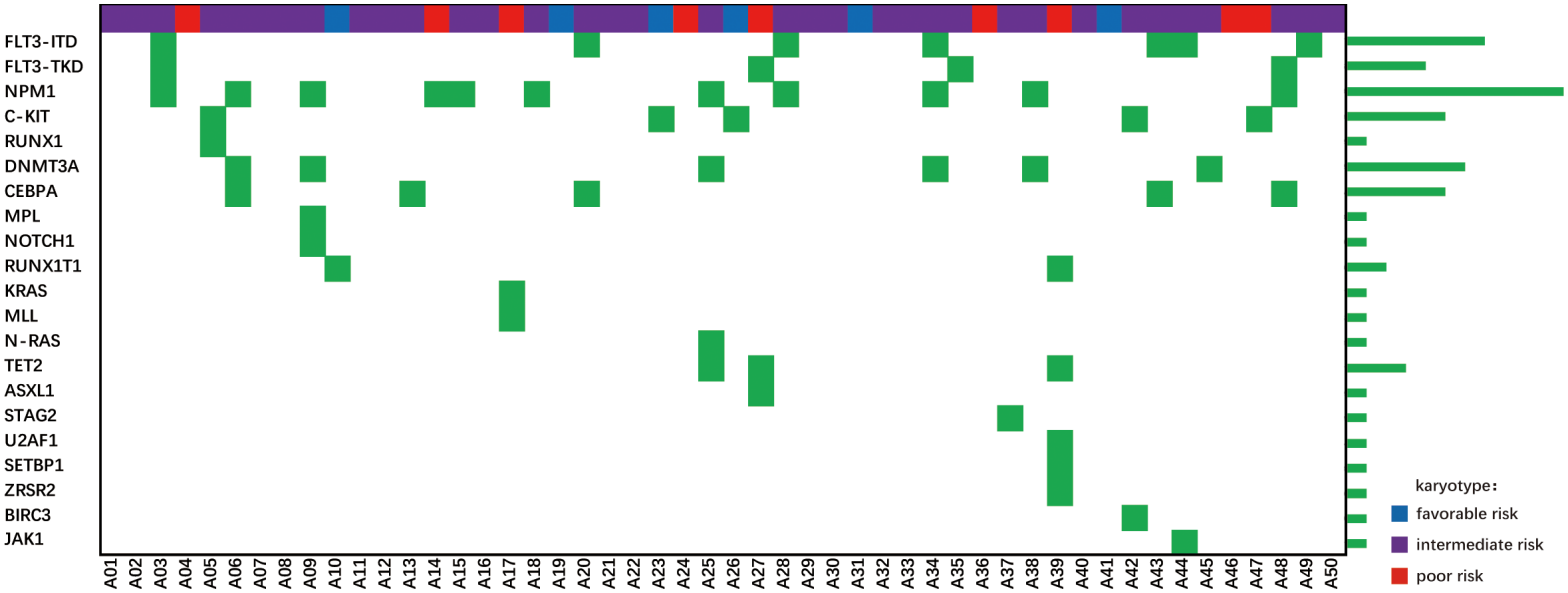
Cytogenetic and next-generation sequencing results of the included patients.

The flow chart of the study is shown in **Figure 1**. Patient demographics and baseline characteristics are summarized in **Table 1**. The median age was 48 years, 10 patients were 60 years or older, and 2 patients were younger than 18 years old. Eighteen patients had leukocyte counts below 4×10^9^/L and 7 patients had leukocyte counts above 100×10^9^/L. Twelve patients were identified as AML with myelodysplasia-related changes based on morphology, cytogenetics and molecular genetics. Eighteen patients had an ECOG score ≥2, and 22 patients had a higher risk of TRM (treatment-related mortality) (20), suggesting that one-third to nearly half of patients are not candidates for intensive chemotherapy. Twenty-six patients received regular consolidation chemotherapy due to age, comorbidities, and lack of suitable transplant donors. Twenty-four patients received allogeneic hematopoietic stem cell transplantation. The cytogenetic and sequencing results of all patients are shown in **Figure 2**. *NPM1* was the most frequently mutated gene (20.0%), followed by *FLT3*-ITD (14.0%), *DNMT3A* (12.0%), *KIT* (10.0%) and *TET2* (6.0%). Six of the patients with high cytogenetic risk had complex karyotypes and three had monosomy karyotypes.

**Table 1:** Baseline characteristics of the patients(n=50).

Characteristics		Number	%
**Age**	Median age (range), years	48 (13-68)	
	<60	40	80.0
	≥60	10	20.0
**Sex**	Male	28	56.0
	Female	22	44.0
**WBC (×10^9^/L)**	<4	18	36.0
	4–100	25	50.0
	>100	7	14.0
**Genetic risk groups**	Favorable risk	8	16.0
	Intermediate risk	29	58.0
	Unfavorable risk	13	26.0
**ECOG PS**	<2	32	64.0
	≥2	18	36.0
**WHO classification**	AML with recurrent genetic abnormalities	23	46.0
	AML with myelodysplasia-related changes	12	24.0
	AML, NOS	13	26.0
	Therapy-related myeloid neoplasms	2	4.0
	Myeloid sarcoma	0	0.0
**Post-treatment**	HSCT	24	48.0
	No-HSCT	26	52.0
**Treatment-related mortality(TRM) risk**	Low (TRM score < 13.1)	28	56.0
	High (TRM score > 13.1)	22	44.0

WBC, white blood cell; ECOG PS, Eastern Cooperative Oncology Group Performance Status; AML, NOS, AML, not otherwise specified; HSCT, hematopoietic stem cell transplantation.

### Efficacy

After one cycle of DAC+HAAG induction therapy, 48 (96%) patients achieved CR/CRp/CRi and 2 (4%) patients achieved PR. Of these two patients, one had AML-M6 with a complex karyotype. The other one had treatment-related AML (secondary to small cell lung cancer with a history of chemotherapy). Both patients achieved CR after a second cycle of reinduction chemotherapy with the DAC+HAAG regimen. Four patients maintained MRD-positive CR, and the remaining 44 patients achieved MRD-negative CR (**Table 2**) All seven patients with *FLT3-*ITD mutations achieved CR.

**Table 2:** Univariable analysis and Multivariable analysis.

Characteristics	OS				PFS			
	Univariable analysis	Multivariable analysis			Univariable analysis	Multivariable analysis	
HR (95% CI)	*P*	HR (95% CI)	*P*	HR (95% CI)	*P*	HR (95% CI)	*P*
**Age (<60)**								
≥60	2.15 (0.88-5.28)	0.094			1.69 (0.71-4.06)	0.238		
**Sex (Male)**								
Female	0.69 (0.29-1.62)	0.397			0.84 (0.38-1.86)	0.669		
**WBC (×10^9^/L) (<4)**								
4–100	1.96 (0.75-5.11)	0.169			1.67 (0.71-3.93)	0.244		
>100	0.92 (0.19-4.58)	0.918			0.71 (0.15-3.38)	0.671		
**Genetics risk groups(Favorable risk)**								
Intermediate risk	1.91 (0.43-8.48)	0.394			0.96 (0.32-2.92)	0.941		
Unfavorable risk	2.59 (0.54-12.54)	0.236			1.17 (0.34-4.00)	0.803		
**ECOG PS (<2)**								
≥2	0.57 (0.22-1.46)	0.242			0.44 (0.17-1.10)	0.080		
**WHO classification (AML with recurrent genetic abnormalities)**								
AML with myelodysplasia-related changes	2.61 (0.94-7.24)	0.065	0.55 (0.16-1.84)	0.331	1.75 (0.69-4.45)	0.238		
AML, NOS	1.05 (0.33-3.33)	0.932	2.67 (0.42-17.08)	0.301	0.68 (0.23-2.00)	0.483		
Therapy-related myeloid neoplasms	9.28 (1.72-50.21)	0.010	7.52 (1.09-52.15)	0.041	4.30 (0.89-20.87)	0.070		
Myeloid sarcoma								
**Post-treatment (HSCT)**								
No-HSCT	4.29 (1.58-11.66)	0.004	5.47 (1.32-22.73)	0.019	2.70 (1.16-6.27)	0.021	2.39 (1.02-5.60)	0.046
**TRM risk (Low (TRM score < 13.1))**								
High (TRM score > 13.1)	27.94 (6.22-125.61)	<0.001	58.77 (7.34-470.78)	<0.001	9.40 (3.54-24.93)	<0.001	8.93 (3.35-23.81)	<0.001

### Overall patient survival

**Figure 3:**
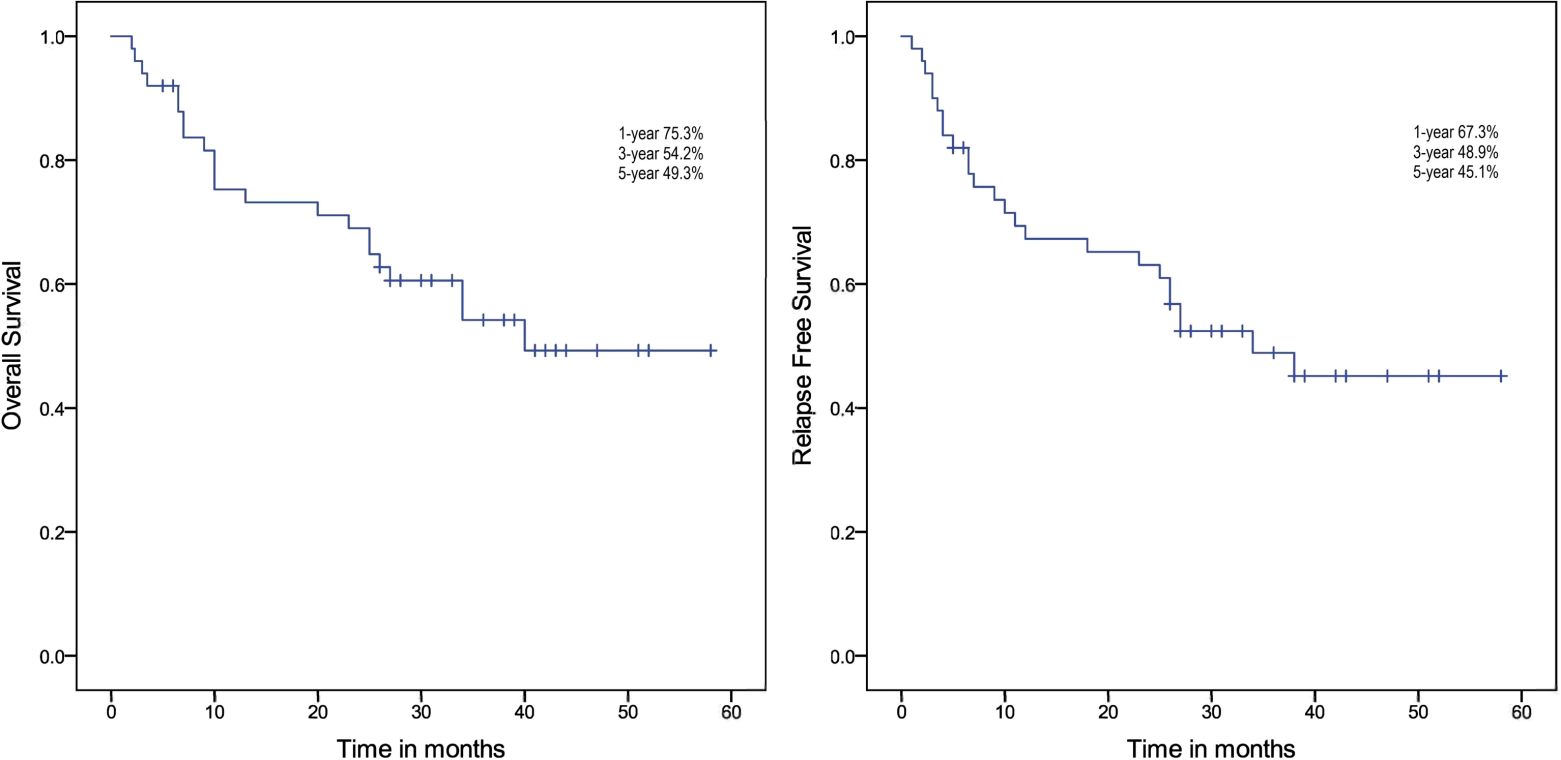
Survival analysis of all the included patients.

Median follow-up was 21.8 months (range 0.5, 58). Four patients were lost to follow-up. Among the 49 CR patients, 25 patients (51.02%) relapsed, including 4 cases (4/8) in the favorable risk group, 14 cases (14/29) in the intermediate risk group, and 7 cases (7/13) in the poor risk group. Median OS was 40.0 months (range 2.0, 58.0). OS at 1, 3, and 5 years were 75.3%, 54.2%, and 49.3%, respectively. The median RFS was 38.0 months (range 2.0, 58.0). The RFS at 1, 3, and 5 years were 67.3%, 48.9%, and 45.1%, respectively (**Figure 3**).

Univariate analysis showed that HSCT, TRM risk and treatment-related AML were the factors influencing OS and RFS. Age, gender, genetic risk and ECOG had no significant effect on prognosis (**Table 3**). Factors with *P*<0.05 in univariate analysis were included in the Cox model, and the results showed that HSCT consolidation (HR,5.47; 95% CI,1.32-22.73, *P*=0.019; HR,2.39; 95% CI,1.02-5.60, *P*=0.046) and TRM risk (HR,58.77; 95% CI,7.34-470.78, *P*<0.001; HR, 8.93; 95% CI,3.35-23.81, *P*<0.001); were independent prognostic factors for improved OS and RFS (**Table 3**).

**Table 3:** Response rates after one cycle of induction therapy(n=50).

Best response	Number	%
CR	40	80.0%
CRp	5	10.0%
CRi	3	6.0%
PR	2	4.0%
ORR	50	100.0%
Early mortality	0	0.0%
MRD negativity at CR/CRp/CRi	44	88.0%

CR, complete remission; CRp, complete remission with incomplete platelet recovery;

CRi, complete remission with incomplete hematologic recovery; PR, partial remission;

ORR, overall response rate; MRD, minimal residual disease.

### Subgroup patient survival

**Figure 4:**
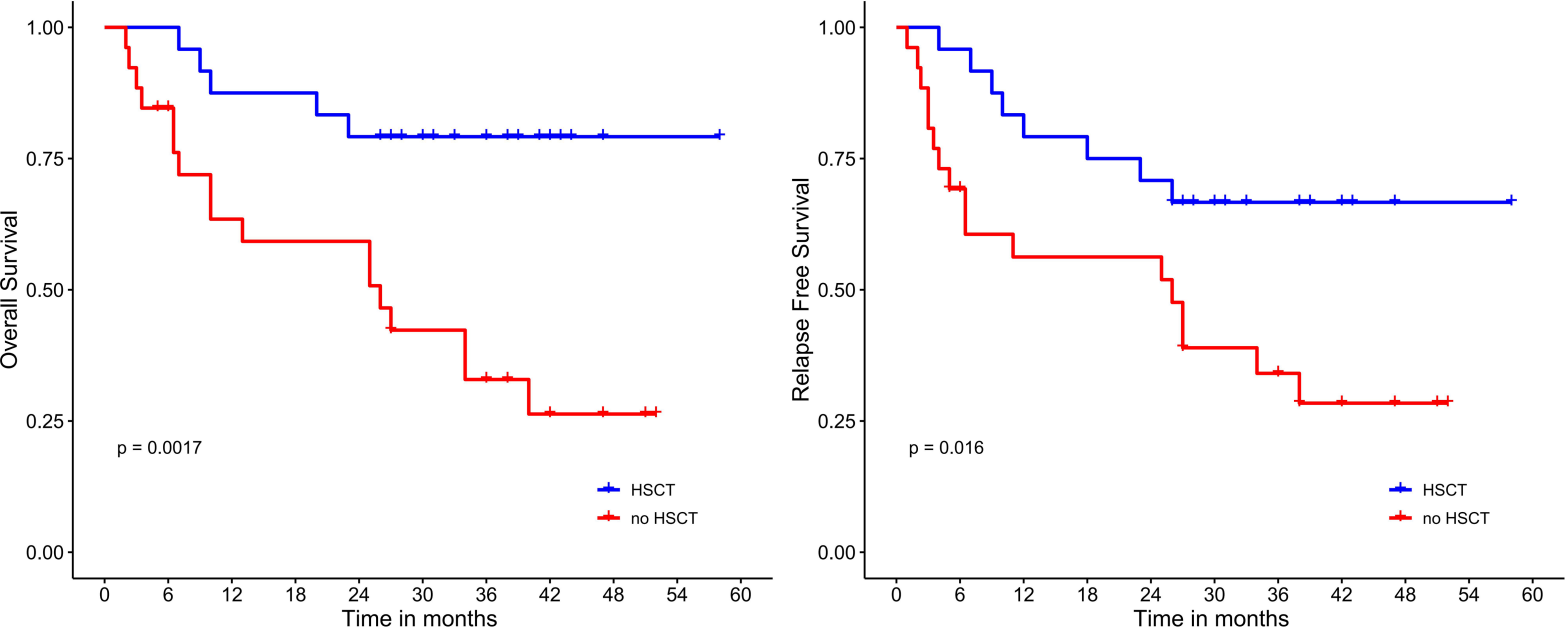
Survival analysis based on HSCT status.

**Figure 5:**
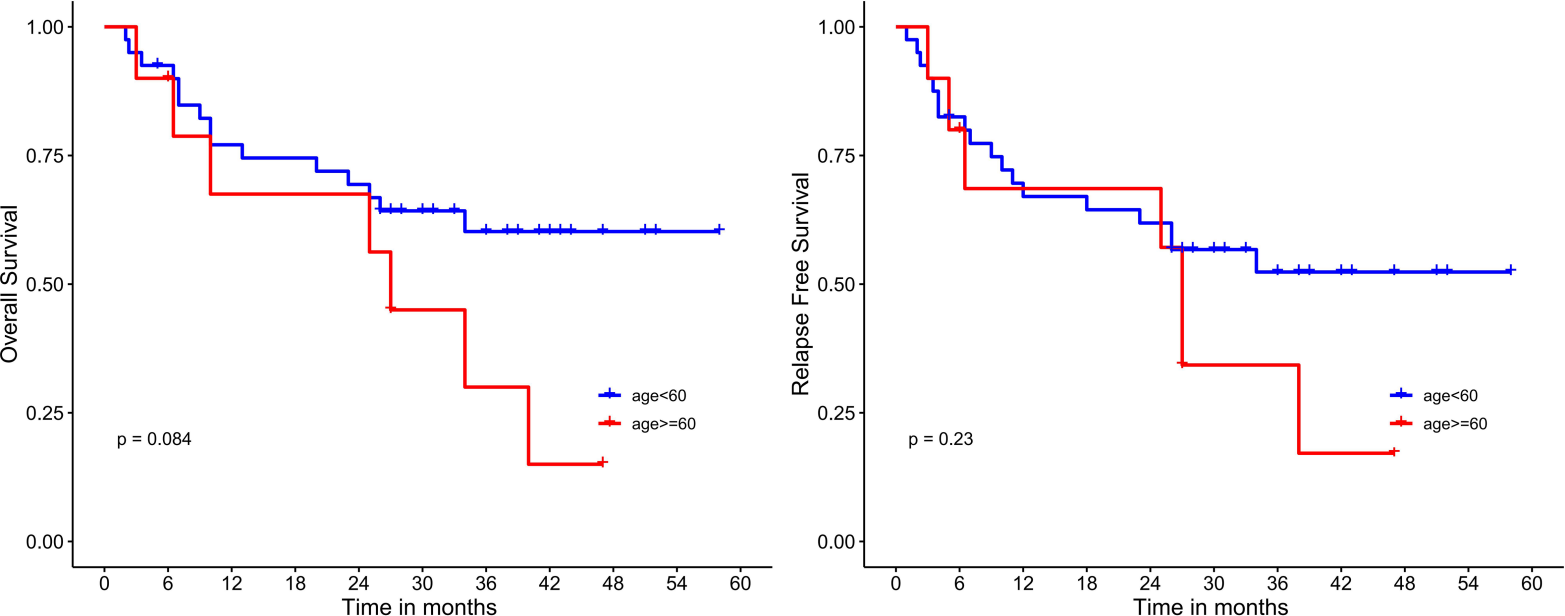
Survival analysis based on age.

**Figure 6:**
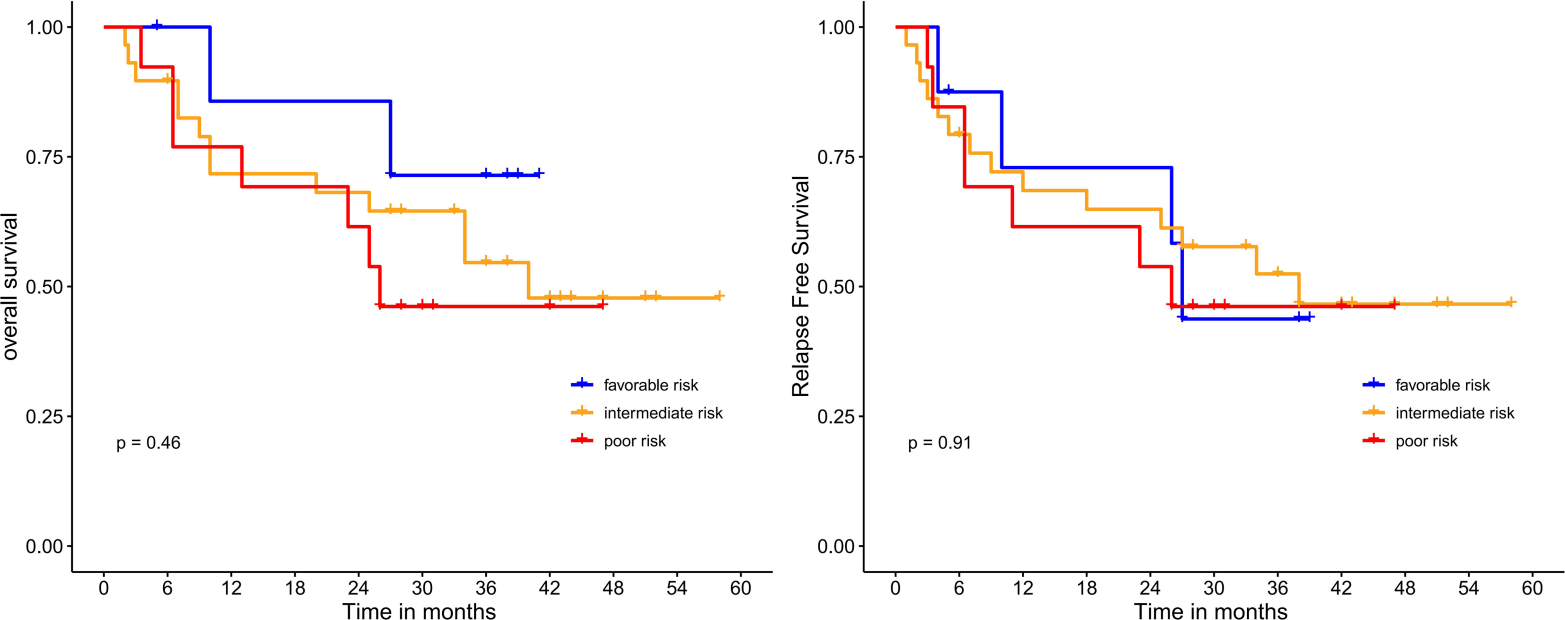
Survival analysis based on genetic risk.

**Figure 7:**
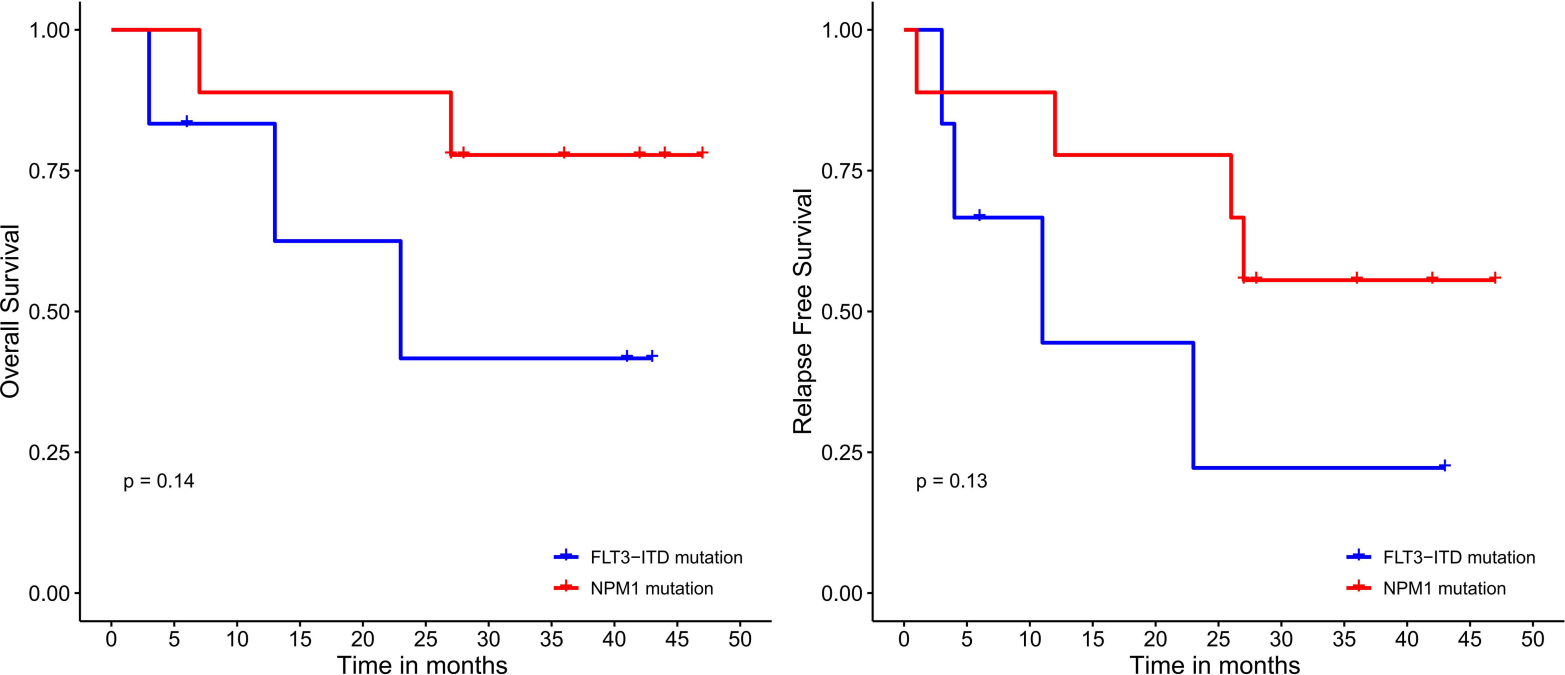
Survival analysis based on *FLT3-ITD* and *NPM1* mutational status.

Median OS and RFS were not reached in patients undergoing HSCT. Meanwhile, among patients who did not receive HSCT, the median OS and RFS were only 26 months (*P*=0.017; 0.016) (**Figure 4**). Median OS and RFS were not reached in patients younger than 60 years. Median OS and RFS for patients aged 60 years or older was only 27 months (*P*=0.084; 0.23) (**Figure 5**). Among patients with different genetic risk groups, the median OS was not reached, 40 months and 26 months in the favorable, intermediate and poor-risk groups (*P*=0.46). The median RFS for the three groups was 27 months, 38 months and 26 months (*P*=0.91) (**Figure 6**). Median OS and RFS were only 23 months and 11 months in patients with *FLT3-*ITD mutations. In contrast, median OS and RFS were not reached in patients with *NPM1* mutations (*P*=0.14; 0.13) (**Figure 7**).

### Safety

The median time to recovery of white blood cell, platelet counts and hemoglobin were 17.5 days (range 0, 32), 19.0 days (range 0, 32) and 20.0 days (range 0, 42), respectively. Different degrees of neutropenia and thrombocytopenia occurred in 100% of patients, but only 16% (8/50) and 6% (3/50) of patients had grade 3-4 infection and bleeding. Non-hematological toxicities were mainly grade 1-2 and resolved after symptomatic treatment (**Table 4**).

**Table 4:** Adverse events (n=50).

Toxicity	Grade 1-2, n (%)	Grade 3-4, n (%)	Total, n (%)
Neutropenia	8 (16)	42 (84)	50 (100)
Thrombocytopenia	6 (12)	44 (88)	50 (100)
Infection	12 (24)	8 (16)	20 (40)
Bleeding	1 (2)	3 (6)	4 (8)
Mucositis/Stomatitis	4 (8)	1 (2)	5 (10)
Hepatotoxicity	7 (4)	1 (2)	8 (16)
Cardiotoxicity	2 (4)	0 (0)	2 (4)

## Discussion

In this study, 18/50 (36%) patients had an ECOG score ≥2, 10/50 (20%) were older than 60 years, and 22/50 (44%) of patients were at high risk for TRM. These patients could not tolerate the toxicity of standard “7+3” regimen. All patients received induction chemotherapy with DAC+HAAG regimen. After one course of chemotherapy, all patients achieved treatment response, and 96.0% of patients attained CR. The response rate of DAC+HAAG is much higher than that reported for other regimens, including CAG, HAG, HCAG and D-CAG (5). Notably, all seven cases with *FLT3-*ITD mutations achieved CR. Among the 7 *FLT3-ITD* patients, 3 had co-mutated *NPM1*, 2 had comutated-biallelic *CEBPA* mutation, and 2 had isolate *FLT3-*ITD mutation. Four of the 7 patients didn’t undergo HSCT because of age and fitness resulting in a worse OS compared with *NPM1* mutation group, but there was no significant difference between the two groups (*P*=0.14, **Figure 7**). In the absence of *FLT3* inhibitors, whether DAC+HAAG regimen is more suitable for patients with *FLT3-ITD* mutations remains to be verified by further expanding the sample size in the future. The OS and RFS of patients who received HSCT were significantly better than those of patients who did not receive HSCT (*p*=0.017; 0.016) (**Figure 4**), suggesting that although DAC+HAAG regimen can achieve very high CR rates, HSCT consolidation is still required to prolong survival. OS was slightly better in patients ≥60 years than in patients under 60 years, mainly because fewer patients received HSCT (≥60 vs < 60 = 0:24).

A meta-analysis of CAG (Ara-C, aclarubicin and G-CSF) for the treatment of 327 newly diagnosed AML patients demonstrated a CR rate of 56.7% (21). And the response rate was significantly higher in patients with favorable (64.5%) and intermediate risk (69.6%) as compared to those with unfavorable risk (29.5%). In a retrospective study, Jin et al. Reported that CAG and IA regimens had similar CR rates in elderly patients with newly diagnosed AML (55.8% *vs*. 52.9%) (22). Homoharringtonine, a plant alkaloid first isolated from Cephalotaxus in China, induces apoptosis in leukemia cells by incorporating into cellular DNA and inhibiting DNA synthesis. It is therapeutically equivalent to aclarubicin, but with less cardiotoxicity. Homoharringtonine has a synergistic anti-leukemia effect in combination with decitabine or doxorubicin (23, 24). Jin et al. reported that HHT could improve the sensitivity to AML treatment by inhibiting the FLT3/MYC pathway (25). CAG or CAG-like regimens are effective in newly diagnosed AML. The addition of homoharringtonine and decitabine has been shown to produce higher response rates. Ye et al. conducted a meta-analysis of the efficacy of HAG (homoharringtonine, Ara-C and G-CSF) regimen in 318 newly diagnosed AML patients and found a CR rate of 62% (5). Zhang et al. conducted a retrospective study of elderly AML patients who had failed a 7 + 3 regimen and showed that patients who received HCAG had a higher ORR than those who received CAG (63.0% *vs.* 43.5%) (26). Decitabine is a 2’-deoxynucleoside analog that specifically inhibits DNA methyltransferase (27), restoring tumor suppressor and DNA repair genes to their normal demethylation state. Decitabine increases the body’s anti-tumor immunity, enhances immune function, and recognizes and kills leukemia cells by depleting Tregs content, thereby promoting remission in patients (28). In addition, decitabine reduces the release of NKG2DL, thereby enhancing the body’s ability to recognize AML blasts (29, 30). In a prospective study of decitabine in combination with a CAG regimen (D-CAG) in elderly patients with newly diagnosed AML, Li et al. showed that 64.7% patients achieved CR after one cycle of therapy, with an ORR of 82.4%, indicating that D-CAG is feasible, safe and effective in elderly AML patents (31).

According to NCCN guideline, the IA regimen is recommended for AML patients eligible for intensive chemotherapy, with an early mortality rate of approximately 15% in most reports. In our study, no patients who received the DAC+HAAG regimen had early death. Although all patients experienced varying degrees of neutropenia and thrombocytopenia, only a minority experienced grade 3-4 bleeding and infection. In terms of non-hematological toxicity, the main adverse events were grade 1-2, which disappeared after symptomatic treatment. Therefore, the DAC+HAAG regimen is safe in newly diagnosed AML. Although the DAC+HAAG regimen was initially designed for elderly/unfit AML patients, results of this study also showed a comparable response rate and survival in patients younger than 60 years old (40/50). It shows that DAC+HAAG regimen is not only suitable for elderly/unfit patients, but also for young/fit patients.

The study has several limitations. This is a retrospective and non-controlled study from a single tertiary center. Further statistical analysis was difficult due to the small sample size. Because of the limited number of patients in this study, the benefit of DAC+HAAG as first-line induction chemotherapy in AML needs to be validated in a prospective randomized controlled study in a larger population.

## Conclusion

In summary, the present findings suggest that the DAC+HAAG regimen is effective and well-tolerated as induction therapy in adult patients with newly-diagnosed AML. There is a reason to believe that this regimen can be used as a first-line induction regimen in patients under 60 years of age. A multicenter randomized controlled trial is current underway to compare the DAC+HAAG and IA regimen (NCT04087967, NCT04083911).

## Data availability statement

The original contributions presented in the study are included in the article/supplementary material. Further inquiries can be directed to the corresponding authors.

## Ethics statement

The protocol was reviewed and approved by the Ethics Committee of the First Affiliated Hospital of Soochow University prior to initiation of the study. The study was conducted in accordance with the ethical principles of the Declaration of Helsinki. All patients provided written informed consent for using and publication of the clinical data.

## Author contributions

J-FZ collected the data, analyzed the results and wrote the manuscript. H-PD treated the patients and revised the manuscript. JY, ZL, Q-YC, X-PT, Z-MJ, and X-MZ treated the patients and collected the data. Q-QZ and S-NL analyzed the data. D-PW and X-WT designed the study, treated the patients and reviewed the manuscript. All authors contributed to the article and approved the submitted version.

## Funding

This study was supported by research grants from National Natural Science Foundation of China (81873443), National Science and Technology Major Project (2017ZX 09304021), National Key R&D Program of China (2016YFC0902800), Priority Academic Program Development of Jiangsu Higher Education Institutions (PAPD), Frontier Clinical Technical Project of the Science and Technology Department of Jiangsu Province(BE2017655), Jiangsu Provincial Medical Talent (ZDRCA2016045), Major Natural Science Research Projects in institutions of higher education of Jiangsu Province (19KJA210002). The Key Science Research Project of Jiangsu Commission of Health (K2019022).

## Acknowledgments

We thank the patients, families, and caregivers who have made the study possible and the clinical study teams.

## Conflict of interest

The authors declare that the research was conducted in the absence of any commercial or financial relationships that could be construed as a potential conflict of interest.

## Publisher’s note

All claims expressed in this article are solely those of the authors and do not necessarily represent those of their affiliated organizations, or those of the publisher, the editors and the reviewers. Any product that may be evaluated in this article, or claim that may be made by its manufacturer, is not guaranteed or endorsed by the publisher.

## References

[1] Döhner H Weisdorf DJ Bloomfield CD. Acute myeloid leukemia. N Engl J Med (2015) 373:1136–52. doi: 10.1056/NEJMra1406184 26376137

[2] Döhner H Estey E Grimwade D Amadori S Appelbaum FR Büchner T. Diagnosis and management of AML in adults: 2017 ELN recommendations from an international expert panel. Blood (2017) 129(4):424–47. doi: 10.1182/blood-2016-08-733196 PMC5291965 27895058

[3] Fernandez HF Sun Z Yao X Litzow MR Luger SM Paietta EM. Anthracycline dose intensification in acute myeloid leukemia. N Engl J Med (2009) 361:1249–59. doi: 10.1056/NEJMoa0904544 PMC4480917 19776406

[4] Yamada K Furusawa S Saito K Waga K Koike T Arimura H. Concurrent use of granulocyte colony-stimulating factor with low-dose cytosine arabinoside and aclarubicin for previously treated acute myelogenous leukemia: a pilot study. Leukemia (1995) 9:10–4. 7531259

[5] Xie M Jiang Q Li L Zhu J Zhu L Zhou D. HAG (Homoharringtonine, cytarabine, G-CSF) regimen for the treatment of acute myeloid leukemia and myelodysplastic syndrome: a meta-analysis with 2,314 participants. PLoS One (2016) 11:e0164238. doi: 10.1371/journal.pone.0164238 27706258 PMC5051946

[6] Przybilla J Hopp L Lübbert M Loeffler M Galle J. Targeting DNA hypermethylation:computational modeling of DNA demethylation treatment of acute myeloid leukemia. Epigenetics (2017) 12(10):886–96. doi: 10.1080/15592294.2017.1361090 PMC5788435 28758855

[7] Cany J Roeven MWH van Evert JSH Hobo W Maas F Fernandez RF. Decitabine enhances targeting of AML cells by CD34(+) progenitor-derived NK cells in NOD/SCID/IL2Rg(null) mice. Blood (2018) 131:202–14. doi: 10.1182/blood-2017-06-790204 PMC5757681 29138222

[8] Dombret H Seymour JF Butrym A Wierzbowska A Selleslag D Jang JH. International phase 3 study of azacitidine vs conventional care regimens in older patients with newly diagnosed AML with >30% blasts. Blood (2015) 126:291–9. doi: 10.1182/blood-2015-01-621664 PMC4504945 25987659

[9] He PF Zhou JD Yao DM Ma JC Wen XM Zhang ZH. Efficacy and safety of decitabine in treatment of elderly patients with acute myeloid leukemia: a systematic review and meta-analysis. Oncotarget (2017) 8:41498–507. doi: 10.18632/oncotarget.17241 PMC5522197 28489568

[10] Li GH Chen RA Ji YR Qin WW Chen Y Wang WQ. Clinical efficacy of decitabine-based chemotherapy regimens in the treatment of newly diagnosed elderly patients with acute myeloid leukemia. Zhongguo Shi Yan Xue Ye Xue Za Zhi (2018) 26:743–9. doi: 10.7534/j.issn.1009-2137.2018.03.019 29950214

[11] Kantarjian H O’Brien S Jabbour E Barnes G Pathak A Cortes J. Effectiveness of homoharringtonine (omacetaxine mepesuccinate) for treatment of acute myeloid leukemia: a meta-analysis of Chinese studies. Clin Lymphoma Myeloma Leuk (2015) 15:13–21. doi: 10.1016/j.clml.2014.09.011 25458084

[12] Chen X-J Zhang W-N Chen B Xi W-D Lu Y Huang J-Y. Homoharringtonine deregulates MYC transcriptional expression by directly binding NF-κB repressing factor. Proc Natl Acad Sci USA (2019) 116(6):2220–5. doi: 10.1073/pnas.1818539116 PMC6369765 30659143

[13] Cao J Feng H Ding NN Wu QY Chen C Niu MS. Homoharringtonine combined with aclarubicin and cytarabine synergistically induces apoptosis in t(8;21) leukemia cells and triggers caspase-3-mediated cleavage of the AML1-ETO oncoprotein. Cancer Med (2016) 5:3205–13. doi: 10.1002/cam4.913 PMC5119976 27709797

[14] Cui W Jin Z Wu D Tang X. Decitabine combined with HAAG regimen is an effective salvage treatment for advanced acute myeloid leukemia. J Third Mil Med Univ (2016) 38:1379–84. doi: 10.16016/j.1000-5404.201601179

[15] Arber DA Orazi A Hasserjian R Thiele J Borowitz MJ Le Beau MM. The 2016 revision to the world health organization classification of myeloid neoplasms and acute leukemia. Blood (2016) 127:2391–405. doi: 10.1182/blood-2016-03-643544 27069254

[16] Li T Xue Y Wu Y Pan J. Clinical and molecular cytogenetic studies in seven patients with myeloid diseases characterized by i(20q-). Br J Haematol (2004) 125(3):337–42. doi: 10.1111/j.1365-2141.2004.04921.x 15086414

[17] Li MM Datto M Duncavage EJ Kulkarni S Lindeman NI Roy S. Standards and guidelines for the interpretation and reporting of sequence variants in cancer: A joint consensus recommendation of the association for molecular pathology, American society of clinical oncology, and college of American pathologists. J Mol Diagn (2016) 19(1):4–23. doi: 10.1016/j.jmoldx.2016.10.002 PMC5707196 27993330

[18] Majhail NS Farnia SH Carpenter PA Champlin RE Crawford S Marks DI. Indications for autologous and allogeneic hematopoietic cell transplantation: guidelines from the american society for blood and marrow transplantation. Biol Blood Marrow Transplant (2015) 21:1863–9. doi: 10.1016/j.bbmt.2015.07.032 PMC4830270 26256941

[19] Cheson BD Bennett JM Kopecky KJ Büchner T Willman CL Estey EH. International working group for diagnosis, standardization of response criteria, treatment outcomes, and reporting standards for therapeutic trials in acute myeloid leukemia. revised recommendations of the international working group for diagnosis, standardization of response criteria, treatment outcomes, and reporting standards for therapeutic trials in acute myeloid leukemia. J Clin Oncol (2003) 21:4642–9. doi: 10.1200/JCO.2003.04.036 14673054

[20] Roland B Megan O Gautam B Hagop A. Prediction of early death after induction therapy for newly diagnosed acute myeloid leukemia with pretreatment risk scores:a novel paradigm for treatment assignment. J Clin Oncol (2011) 29(33):4417–24. doi: 10.1200/JCO.2011.35.7525 PMC3221524 21969499

[21] Wei G Ni W Chiao JW Cai Z Huang H Liu D. A meta-analysis of CAG (cytarabine, aclarubicin, G-CSF) regimen for the treatment of 1029 patients with acute myeloid leukemia and myelodysplastic syndrome. J Hematol Oncol (2011) 4:46. doi: 10.1186/1756-8722-4-46 22082134 PMC3230125

[22] Jin J Chen J Suo S Qian W Meng H Mai W. Low-dose cytarabine, aclarubicin and granulocyte colony-stimulating factor priming regimen versus idarubicin plus cytarabine regimen as induction therapy for older patients with acute myeloid leukemia. Leuk Lymphoma (2015) 56:1691–7. doi: 10.3109/10428194.2014.963074 25257348

[23] Geng S Yao H Weng J Tong J Huang X Wu P. Effects of the combination of decitabine and homoharringtonine in SKM-1 and kg-1a cells. Leuk Res (2016) 44:17–24. doi: 10.1016/j.leukres.2016.02.002 26991610

[24] Yan D Wei H Lai X Ge Y Xu S Meng J. Co-Delivery of homoharringtonine and doxorubicin boosts therapeutic efficacy of refractory acute myeloid leukemia. J Control Release (2020) 10:327:766–78. doi: 10.1016/j.jconrel.2020.09.031 32949646

[25] Li C Dong L Su R Bi Y Qing Y Deng X. Homoharringtonine exhibits potent anti-tumor effect and modulates DNA epigenome in acute myeloid leukemia by targeting SP1/TET1/5hmC. Haematologica (2018) 105(1):148–60. doi: 10.3324/haematol.2018.208835 PMC6939512 30975912

[26] Zhang JY Li L Liu W Jin Y Zhao M Zhou Y. Comparison of efficacy of HCAG and CAG re-induction chemotherapy in elderly low- and intermediate-risk group patients diagnosed with acute myeloid leukemia. Clin Transl Oncol (2020) 23:48–57. doi: 10.1007/s12094-020-02383-x 32458310

[27] Sorm F Pískala A Cihák A Veselý J. 5-azacytidine, a new, highly effective cancerostatic. Experientia (1964) 20:202–3. doi: 10.1007/bf02135399 5322617

[28] Hu R Gao Y Wen Y Wu K Duan C Zeng Y. Effect of decitabine on regulatory T cells relative content in peripheral blood and bone marrow of patients with myelodysplastic Syndrome/Acute myeloid leukemia. Zhongguo Shi Yan Xue Ye Xue Za Zhi (2022) 30(1):36–42. doi: 10.19746/j.cnki.issn.1009-2137.2022.01.007 35123601

[29] Raneros AB Palanco VM Fernandez AF Rodriguez RM Fraga MF Lopez-Larrea C. Methylation of NKG2D ligands contributes to immune system evasion in acute myeloid leukemia. Genes Immun (2014) 16(1):71–82. doi: 10.1038/gene.2014.58 25393931

[30] Baragano Raneros A Minguela A Rodriguez RM Colado E Bernal T Anguita E. Increasing TIMP3 expression by hypomethylating agents diminishes soluble MICA, MICB and ULBP2 shedding in acute myeloid leukemia, facilitating NK cell-mediated immune recognition. Oncotarget (2017) 8(19):31959–76. doi: 10.18632/oncotarget.16657 PMC5458262 28404876

[31] Li J Chen Y Zhu Y Zhou J Xu Y Li Y. Efficacy and safety of decitabine in combination with G-CSF, low-dose cytarabine and aclarubicin in newly diagnosed elderly patients with acute myeloid leukemia. Oncotarget (2015) 6:6448–58. doi: 10.18632/oncotarget.3361 PMC4467448 25749041

